# Pancreatic Tuberculosis: A Diagnostic Dilemma

**DOI:** 10.7759/cureus.16734

**Published:** 2021-07-29

**Authors:** Shahan Haseeb, Muhammad I Bilal, Syed A Bokhari, Rida T Mirza, Shahzad Riyaz

**Affiliations:** 1 Internal Medicine, Shifa International Hospital Islamabad, Islamabad, PAK; 2 Medicine, Shifa College of Medicine, Islamabad, Islamabad, PAK; 3 General surgery, Shifa International Hospital Islamabad, Islamabad, PAK; 4 Gastroenterology, Shifa International Hospital Islamabad, Islamabad, PAK

**Keywords:** pancreatic tuberculosis, endoscopic ultrasound, tuberculosis, incidental diagnosis, tb

## Abstract

Despite the high prevalence of tuberculosis (TB) in developing countries, pancreatic TB remains a rare disease. Pancreatic TB usually presents as fever, night sweats, and abdominal pain in an immunocompromised individual. We present a case of a patient with end-stage renal disease undergoing pre-transplant workup who had an incidental finding of a pancreatic mass and necrotic peri-pancreatic lymph nodes on a CT scan. The patient was diagnosed via endoscopic ultrasound-guided biopsy as pancreatic TB. Anti-TB therapy was started with positive results.

## Introduction

Even in parts of the world where tuberculosis (TB) is highly prevalent, isolated pancreatic TB is a rare entity, often presenting as a diagnostic challenge [1]. Pancreatic TB commonly presents as a mass lesion causing compression of local structures and mimics pancreatic malignancy, both clinically and radiologically [2]. Endoscopic ultrasound with fine-needle aspiration (EUS-FNA) can provide an accurate diagnosis [1]. Often the diagnosis is made postoperatively (on histopathology after pancreatic mass removal) due to the low index of suspicion of this entity and masquerading of its symptoms as more common pancreatic conditions. We present a case of an incidental finding of a pancreatic mass which was diagnosed and treated correctly as isolated pancreatic TB.

## Case presentation

A 50-year-old male was referred to the Nephrology team for a renal transplant workup in March 2020. He had a history of diabetes and hypertension. He was diagnosed with obstructive uropathy in 2013 and subsequently developed end-stage renal disease with a baseline creatinine of three. He was managed successfully with medication till March 2020 when his renal functions deteriorated and he was offered a renal transplant.

**Figure 1:**
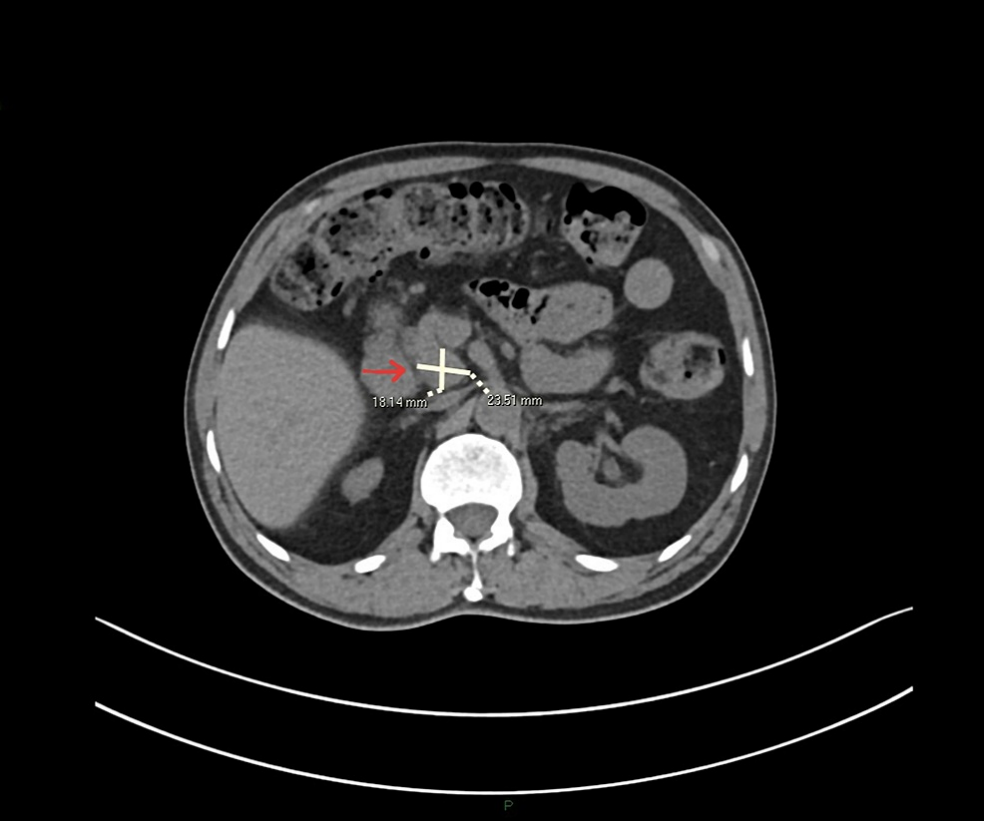
CT abdomen. Peri pancreatic lymph nodes (arrow) on initial CT scan performed during pre-transplant workup.

As part of the pre-transplant workup, a CT scan of the abdomen, chest, and pelvis suggested multiple enlarged necrotic lymph nodes in the peripancreatic and portocaval areas. The largest node was found abutting the head of the pancreas measuring 23 mm x 18 mm (Figure 1). The patient’s hemoglobin, white blood count, and C-reactive protein levels were 8.9 g/dL, 12,000/mm3, and 7.63 mg/L. The liver function tests were within the normal range. Unfortunately, the patient was lost to follow up.

The patient was re-admitted via the emergency department (ED) in December 2020 with fever, decreased urine output, and right sub-costal pain. Initial investigations revealed hemoglobin, white blood count, and C-reactive protein levels of 6.7 g/dL, 16,000/mm3, and 65.88 mg/L respectively. Liver function tests were normal. A CT scan of the abdomen suggested an interval increase in the size of the peripancreatic and portocaval lymph nodes. The patient's chest X-ray was performed, which was unremarkable.

**Figure 2:**
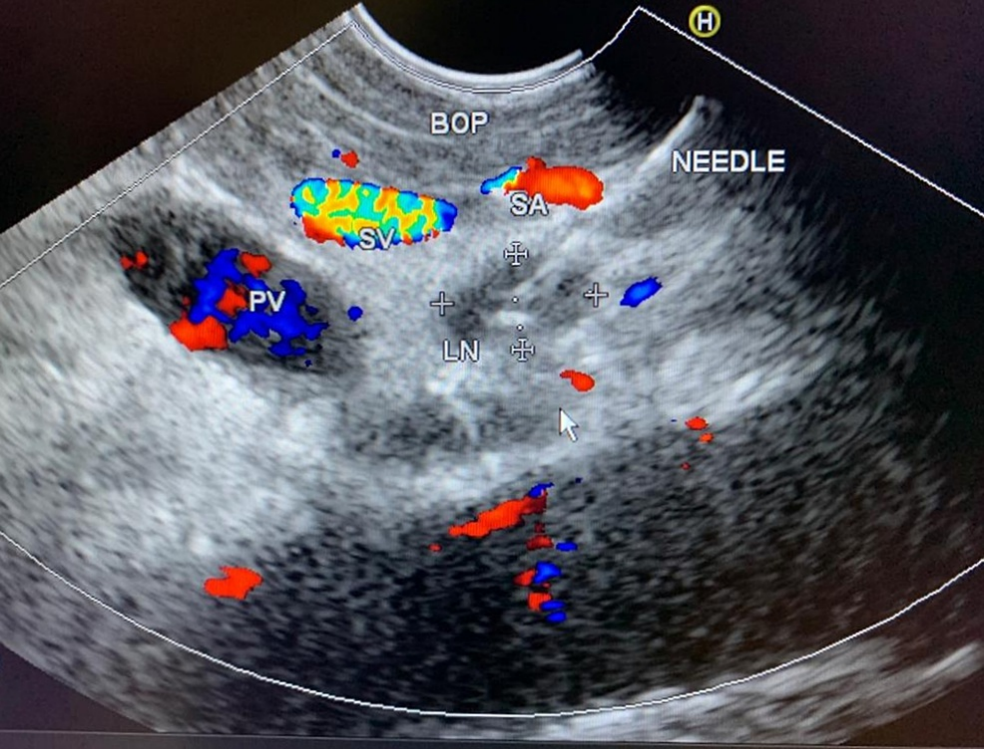
Endoscopic ultrasound. PV, portal vein; BOP, body of pancreas; LN, lymph node; SV, splenic vein; SA, splenic artery

**Figure 3:**
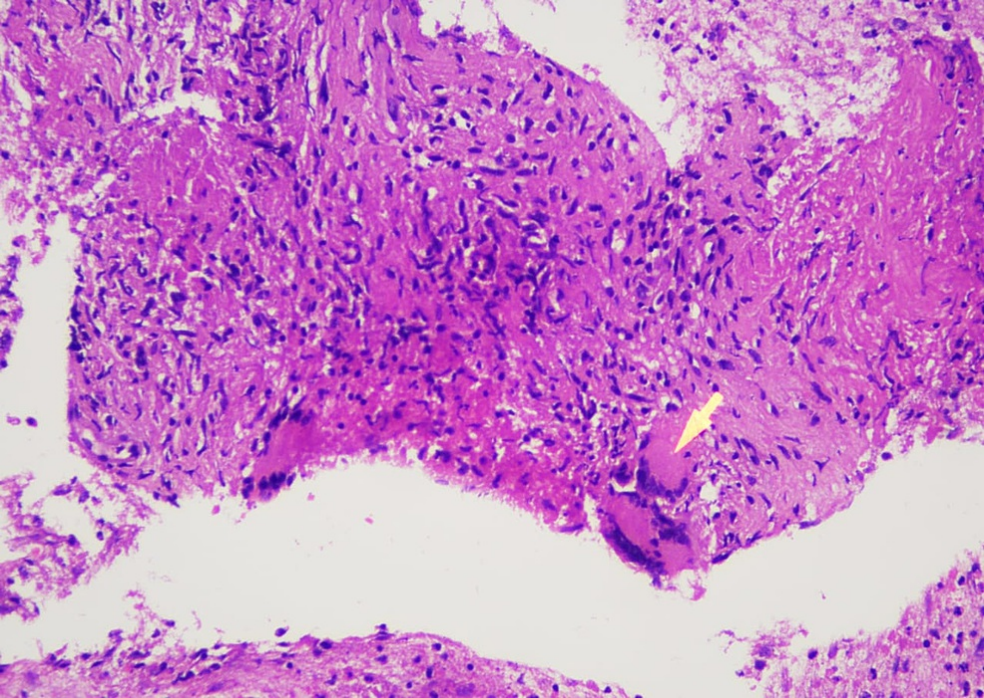
Endoscopic ultrasound-guided biopsy. Epithelioid histiocyte (arrow) forming granulomas. Characteristic of TB. TB, tuberculosis

He was subsequently referred for endoscopic ultrasound (EUS) guided biopsy of the peripancreatic lymph node mass. EUS was performed with a curvilinear scope. A necrotic lymph node mass was identified near the uncinate/head of the pancreas (Figure 2). Multiple biopsies were taken with a 22G FNB Acquire needle. Histopathology suggested the presence of predominantly necrotic and scanty fibro collagenous tissue containing occasional granulomas. The granulomas were composed of epithelioid histiocytes and giant cells (Figure 3). Mycobacterium tuberculosis-polymerase chain reaction (MTB-PCR) was positive confirming the diagnosis of TB.

**Figure 4:**
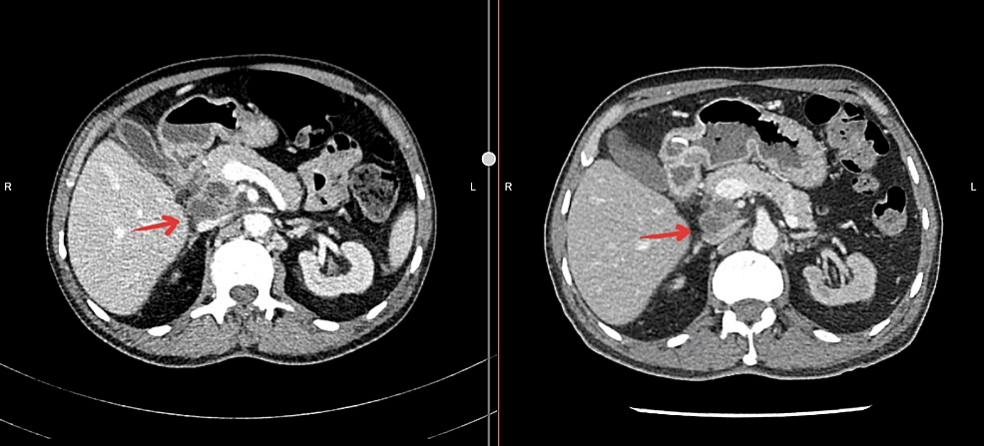
Comparison of CT abdomen performed before (Left) and after (Right) initiation of treatment. Significant reduction in the size of the central necrotic lymph nodes, three months after the initiation of antitubercular therapy.

The patient was commenced on anti-tuberculosis treatment. A repeat CT abdomen in March 2021 suggested interval reduction in the sizes of the centrally necrotic lymph nodes in peripancreatic and portocaval locations compared to the CT abdomen performed in December 2020, from 47 mm x 26 mm to 35 mm x 30 mm and 26 mm x 35 mm to 18 mm x 23 mm respectively (Figure 4). Clinically the patient had improved significantly with no further episodes of pyrexia or abdominal pain. The blood tests improved as well, with hemoglobin, white blood count, and C-reactive protein levels of 13.9 g/dL, 8500/mm3, and 2.02 mg/L respectively. Renal transplantation is planned once the patient completes his treatment for TB.

## Discussion

Primary pancreatic TB (PPTB) is an isolated involvement of the pancreas or peripancreatic lymph nodes by TB in the absence of previously identified TB and involvement of any other organ [2]. Owing to the hidden location of the pancreas in the retroperitoneum along with the antimycobacterial properties of its enzymes, pancreatic TB is a rare entity [3-4]. It manifests itself, both clinically and radiologically, as a pancreatic malignancy. Consequently, the diagnosis of pancreatic TB is considerably difficult, and most of the reported cases were diagnosed after surgical intervention for suspected pancreatic malignancy [3]. Pancreatic involvement in TB is most commonly a part of military TB [5].

Patients with pancreatic TB can remain asymptomatic initially and later present with non-specific complaints [5]. The clinical symptomatology can be variable and include abdominal pain, anorexia, weight loss, and jaundice; possibly associated with fever and night sweats, palpable epigastric mass, and peripheral lymphadenopathy. Jaundice has been reported in three out of four of these patients and is likely due to bile duct obstruction caused by pancreatic mass or peripancreatic lymph nodes. Gastrointestinal bleeding may occur because of the formation of arterial pseudoaneurysms, portal hypertension, or duodenal mucosal involvement. Other complications include gastric outlet obstruction, portal hypertension, diabetes mellitus, abscess formation, and recurrent acute pancreatitis [6]. Pancreatic TB may also present with peri-pancreatic lymph node enlargement and inflammation [7].

There are no radiologic features specific to pancreatic TB. Typically, pancreatic lesions from TB are heterogeneous and multi-cystic on imaging. CT findings may include hypodense lesions with irregular borders most commonly in the head of the pancreas, or enlarged peripancreatic lymph nodes [8]. Findings that may suggest mycobacterial infection include the presence of rim-enhanced lymph nodes in the peripancreatic region and a thickened bowel wall in the ileocecal region [4]. An important image finding in pancreatic TB is the normal-appearing common bile duct and the pancreatic duct, regardless of the location of the mass. This is in contrast to pancreatic adenocarcinoma where the pancreatic duct is dilated in tumors of the head region [9]. However, given the lack of pathognomonic findings on imaging, the diagnosis of pancreatic TB requires a histological/cytological diagnosis [8].

The technique of biopsy includes an endoscopic ultrasound (US)-guided biopsy, CT/US-guided percutaneous biopsy, and surgical biopsy (open or laparoscopic). The unique microscopic features are those of caseating granulomatous inflammation. Acid-fast bacilli may also be found in roughly 35% of cases [2]. The treatment of pancreatic TB is the same as any other type of extrapulmonary TB. Standard anti-tuberculous therapy involving the use of at least four drugs remains the cornerstone of treatment. Patients with pancreatic TB respond very well to conventional anti-tuberculous treatment. Reported literature recommends the use of anti-tuberculous drugs for 6-12 months. Isoniazid rifampicin, pyrazinamide, and ethambutol are used for two to four months with subsequent isoniazid and rifampicin for 6-12 months [2, 5].

## Conclusions

There were some interesting aspects to our case. Despite being an immunosuppressed individual (end-stage renal disease and history of uncontrolled diabetes mellitus) there were no symptoms of weight loss, anorexia, fever, epigastric pain, or night sweats typical of pancreatic TB. The patient’s diagnosis was based solely on an incidental finding. The discovery of pancreatic TB on EUS-guided biopsy was a game-changer in our case as it allowed the patient to be considered for transplant soon after the anti-TB therapy is completed. Had we considered the pancreatic mass as the more common possibility of pancreatic cancer, the patient would have had to undergo major surgery and long-term surveillance and may not have been considered a candidate for his much-needed renal transplant. 
